# Comparative Profiles of the WATCHMAN™ and Amplatzer™ Cardiac Plug/Amplatzer™ Amulet™ Devices for Left Atrial Appendage Closure in Non-valvular Atrial Fibrillation: A Comprehensive Systematic Review and Meta-analysis

**DOI:** 10.19102/icrm.2024.15061

**Published:** 2024-06-15

**Authors:** Fnu Raja, Khimya Rani, Sunny Kumar, Fnu Someshwar, Muhammad Ahsan Naseer Khan, Fnu Abubakar, Dhvani Bhatt, Deepak Jung Subedi, Sujeet Shadmani, Fatima Tuz Zahra Abdullah

**Affiliations:** 1 Department of Internal Medicine, Federal Medical and Dental College, Islamabad, Pakistan; 2 Department of Internal Medicine, Chandka Medical College SMBBMU, Larkana, Pakistan; 3 Department of Internal Medicine, Liaquat National Hospital and Medical College, Karachi, Pakistan; 4 Department of Internal Medicine, King Edward Medical University, Lahore, Pakistan; 5 Department of Internal Medicine, American University of Barbados, Bridgetown, Barbados; 6 Department of Internal Medicine, College of Medical Sciences, Bharatpur, Nepal; 7 Department of Internal Medicine, Shaheed Mohtarma Benazir Bhutto Medical College, Karachi, Pakistan; 8 Department of Internal Medicine, Allama Iqbal Medical College, Lahore, Pakistan

**Keywords:** Amulet™ device, atrial fibrillation, left atrial appendage closure, meta-analysis, WATCHMAN™ device

## Abstract

Atrial fibrillation (AF) is a prevalent cardiac arrhythmia marked by irregular and frequent tachycardic rhythms in the atria, affecting 1%–2% of the general population. The WATCHMAN™ device from Boston Scientific (Marlborough, MA, USA) and the Amplatzer™ Amulet™ device from Abbott (Chicago, IL, USA) are two devices used globally for left atrial appendage closure (LAAC) in non-valvular AF. A systematic search was conducted in PubMed, the Cochrane Library, and Elsevier’s ScienceDirect literature databases to identify studies comparing the WATCHMAN™ procedure with Amulet™ device implantation for LAAC in patients with AF. The analyses were conducted using the random-effects model. A total of 20 studies were identified, with 18 falling into the category of observational studies and 2 being randomized controlled trials. A total of 6310 participants were included in this meta-analysis, with 3198 individuals (50.68%) assigned to the WATCHMAN™ procedure group and 3112 individuals (49.32%) allocated to the Amplatzer™ Cardiac Plug (ACP) group. The analysis revealed a higher risk of stroke associated with the WATCHMAN™ technique (relative risk [RR], 1.14), albeit without statistical significance. Conversely, the WATCHMAN™ approach led to a significantly lower risk of cardiac death (RR, 0.44; *P* = .04). Notably, the risks of all-cause mortality (RR, 0.89; 95% confidence interval [CI], 0.73–1.08; *I*^2^ = 0%; *P* = .25) and major bleeding (RR, 0.93; 95% CI, 0.65–1.33; *I*^2^ = 31%; *P* = .70) were clinically reduced with the WATCHMAN™ procedure, although statistical significance was not achieved. Compared to Amulet™ device implantation, WATCHMAN™ device implantation decreased the risk of cardiac mortality, while the risks of stroke, systemic embolism, all-cause mortality, and major bleeding were not statistically significant.

## Introduction

Atrial fibrillation (AF) is a common heart rhythm disorder characterized by irregular and rapid beating in the atria. It affects 1%–2% of the general population, 6% of those aged ≥65 years, and 13% of individuals aged >80 years.^1^ The global incidence and prevalence of AF are rising, having quadrupled in the last 50 years, according to the Framingham Heart Study.^2^ The left atrial appendage (LAA) is the primary site of embolism in about 90% of non-rheumatic AF-related strokes. This is attributed to the lack of contractile function, leading to blood stasis and thrombus formation. As a result, individuals with AF have a more than fivefold increase in the annual risk of significant stroke compared to the general population.^3^

LAA closure (LAAC) is a non-pharmacological method for lowering the risk of cardioembolic events in AF patients who are at high risk of stroke.^4^ While oral anticoagulation can reduce the risk of cardioembolism by approximately 70% when used concurrently, such has been related to an elevated risk of severe bleeding.^5^ The WATCHMAN™ device and the Amplatzer™ Amulet™ are popular LAAC devices.^5^ Following the Amulet™ IDE trial, the U.S. Food and Drug Administration recently approved the Amplatzer™ Amulet™. Research demonstrated its superiority in LAA occlusion (LAAO), particularly in minimizing leaks >0.5 mm.^6^ Comparing the Amulet™ to the previous-generation WATCHMAN™ 2.5 device revealed this superiority. At the same time, it is important to note that the most recent iteration, WATCHMAN™ FLX, demands consideration in current clinical practice due to significant architectural enhancements.^6^ In observational studies, the Amplatzer™ Cardiac Plug (ACP) and second-generation Amplatzer™ Amulet™ devices have been associated with positive outcomes.^7^ The WATCHMAN™ device reduces the risk of blood clotting and strokes by obstructing the LAA. It includes a self-expanding nickel–titanium frame enveloped in a permeable fabric placed at the ostium of the LAA through a minimally invasive transseptal approach.^8^ In contrast, the Amulet™, featuring a lobe-and-disk mechanism, enhances LAA sealing and coverage.^8,9^

Guidelines recommend these devices for AF patients with a significant stroke risk contraindicated for long-term anticoagulation, having subjected both to rigorous clinical evaluation.^10^ Despite the publication of observational studies and randomized controlled trials (RCTs), conflicting findings and inadequately conducted analyses—particularly regarding infrequent events like stroke—have been documented. This meta-analysis aims to comprehensively compare the safety and effectiveness of the Amplatzer™ and WATCHMAN™ devices. The fundamental differences in design, deployment methods, and clinical outcomes warrant this thorough comparison. While both devices share the same therapeutic goal, their distinct characteristics may lead to variations in efficacy, safety, and compatibility with patient anatomies. The study analyzes data from RCTs, observational studies, and registries to assess stroke-prevention effectiveness, procedural success, complication rates, and long-term outcomes. Through an in-depth analysis of the merits and demerits of each device, our goal was to address the current knowledge gaps and provide accurate suggestions for device selection.

## Methodology

This meta-analysis rigorously adheres to the guidelines outlined in the Preferred Reporting Items for Systematic Reviews and Meta-analyses (PRISMA) statement^11^ to ensure methodological transparency and reliability.

### Data sources and search strategy

A systematic and exhaustive search strategy was implemented to identify relevant studies. Electronic databases, including PubMed, the Cochrane Library, and Elsevier’s ScienceDirect, were systemically searched until December 2023 without language restrictions. The search strategy employed a combination of Medical Subject Heading (MeSH) terms and keywords, such as “Watchman device,” “ACP device,” “Amulet device,” “left atrial appendage closure,” and “non-valvular atrial fibrillation.” The detailed search strategy is summarized in **Supplementary Table S1**.

### Study selection

During study selection, a stringent and comprehensive approach was adopted. Two independent reviewers scrutinized the titles and abstracts of identified studies and evaluated their full texts. Any inconsistencies or disparities in the inclusion of studies were addressed through extensive deliberation and, when necessary, consultation with a third reviewer. The predefined inclusion criteria were rigorously adhered to throughout this selection process. Specifically, the following studies were considered for inclusion: first, studies directly comparing the clinical effectiveness and safety profiles of the WATCHMAN™ device against the ACP/Amulet™ device, specifically in the context of percutaneous LAAC, among patients diagnosed with non-valvular AF; second, studies that encompassed diverse research designs, including RCTs, prospective or retrospective cohort studies, case–control studies, and comparative observational studies; and third, studies that included comprehensive reporting of clinically relevant outcomes, encompassing procedural success rates, adverse events, and long-term follow-up data. This stringent selection process aimed to ensure the incorporation of high-quality and pertinent studies that directly addressed these devices’ comparative efficacy and safety in managing non-valvular AF through percutaneous LAAC.

### Data extraction

Two reviewers performed data extraction independently using a standardized data-extraction form. Extracted data included study characteristics (eg, author, publication year, study design), participant demographics, intervention details, procedural success rates, clinical outcomes (eg, stroke, bleeding events), and adverse events. The outcomes were divided into primary outcomes and secondary outcomes. The primary outcomes included stroke, systemic embolism, all-cause mortality, cardiac death, and major bleeding. Meanwhile, the secondary outcomes included device-related thrombus, peri-device leak (PDL), procedure-related complications, and procedural success. Discrepancies were resolved through discussion or by involving a third reviewer when necessary.

### Quality assessment

The methodological quality and risk of bias of included studies were assessed independently by two reviewers using appropriate tools, such as the Cochrane risk-of-bias tool for RCTs^12^ and the Newcastle–Ottawa scale^13^ for observational studies. Quality assessment covered aspects related to study design, participant selection, blinding, outcome reporting, and other relevant parameters.

### Data synthesis and analysis

The statistical analysis of this meta-analysis was conducted using Review Manager version 5.4.1, a tool developed by The Nordic Cochrane Centre in collaboration with The Cochrane Collaboration in Denmark in 2014. Only comparative studies were included in the analysis. The results were presented through forest plots that display the pooled effect of relative risks (RRs) for dichotomous outcomes and weighted mean differences for continuous outcomes. To ensure the accuracy of the findings, the generic-inverse variance with a random-effects model was used.

The *P* values were set at <.05 to determine the significance of the results. To assess the possibility of publication bias, funnel plots were created for each primary outcome. Higgins’ *I*^2^ test was used to determine the level of heterogeneity, which was categorized as low, moderate, or high. When substantial heterogeneity (>75%)^14^ was detected, a sensitivity analysis was conducted by omitting one study at a time to identify the impact of individual studies on the overall findings.

All analyses were deemed significant if *P* < .05, and the authors conducted a comprehensive analysis of the data to ensure the accuracy and reliability of their findings.

### Ethical considerations

Ethical approval was not required because this meta-analysis involved synthesizing and analyzing previously published data without directly involving human subjects. All data were extracted from publicly available sources with no identifiable patient information, ensuring compliance with ethical guidelines.

## Results

### Eligible studies

**Figure 1:**
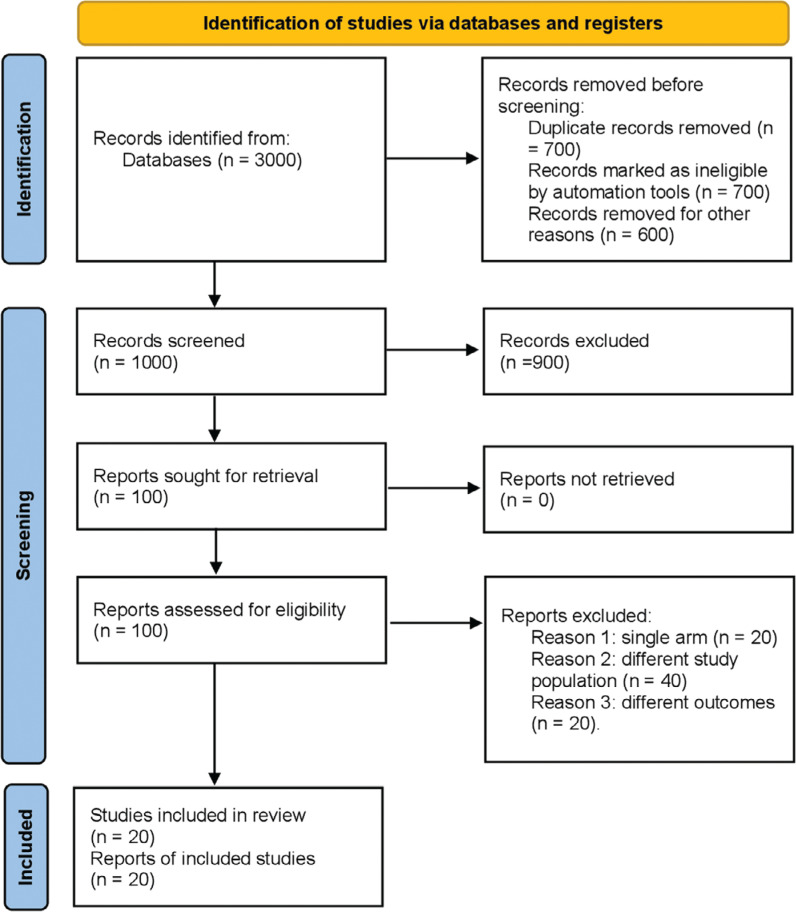
Preferred Reporting Items for Systematic Reviews and Meta-analyses flow diagram illustrating the search strategy and study-selection process for the meta-analysis. The initial search yielded 3000 articles, which were screened for duplicates and evaluated with regard to title and abstract. Twenty studies, including 2 randomized controlled trials and 18 observational studies, met the inclusion criteria for the analysis, with only comparative studies being included.

The initial output of the literature search totaled 3000 articles. Following the removal of duplicates and scrutiny of titles and abstracts, a total of 20 relevant studies^15–34^ were discerned, with 18 falling into the category of observational studies^15–26,29–34^ and 2 being RCTs.^27,28^ Only comparative studies were considered for inclusion in the ensuing meta-analysis. The PRISMA diagram in **Figure 1** illustrates the meticulous search strategy employed. All RCTs were characterized by a comparative, multicenter, and double-blind design. Ten studies within the subset of 18 observational studies were prospective, while the remaining 8 were retrospective. The temporal scope of these publications spanned from 2013–2023.

### Baseline characteristics of the included patients

A total of 6310 participants were included in this meta-analysis, with 3198 individuals (50.68%) assigned to the WATCHMAN™ procedure group and 3112 individuals (49.32%) allocated to the ACP group. The total patient cohort included 3657 women (57.9%). The mean age of all participants was 75 ± 6.3 years. The average CHA_2_DS_2_-VASc score was 4.12 ± 1.3 points, while the average HAS-BLED score was 3.4 ± 1.0 points. Examination of the baseline data revealed a high prevalence of diabetes, hypertension, and a prior history of myocardial infarction among the included individuals. Paroxysmal AF was shown to be the most common kind of AF. **Table 1** provides a comprehensive overview of the baseline characteristics of the patients enrolled in the study.

### Quality assessment and publication bias

**Supplementary Figure S1:**
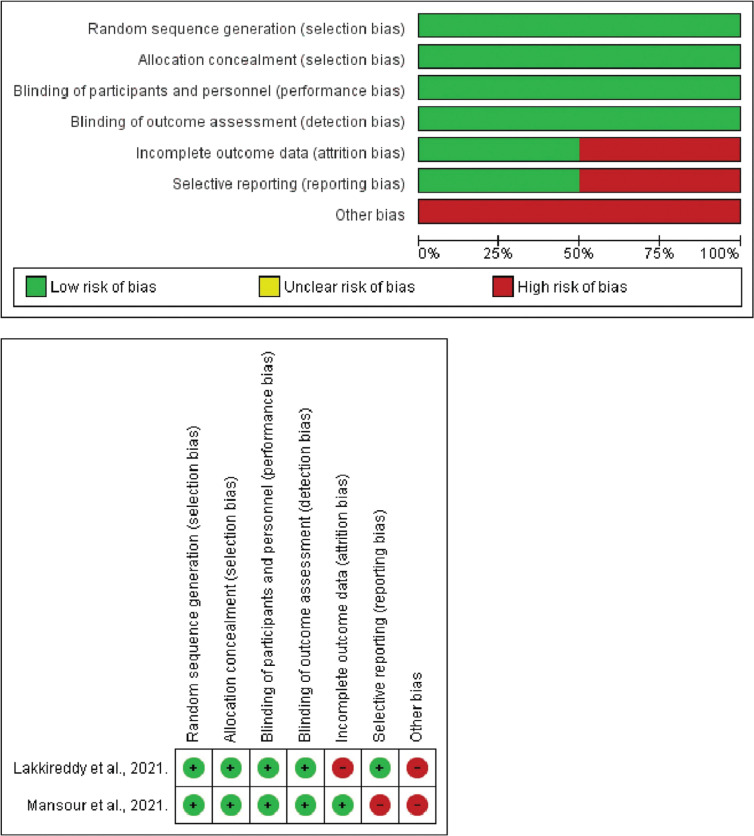
Quality assessment of the included randomized controlled trials. The trials of medium to high quality were identified using the Cochrane method for assessing randomized controlled trials.

**Supplementary Figure S2:**
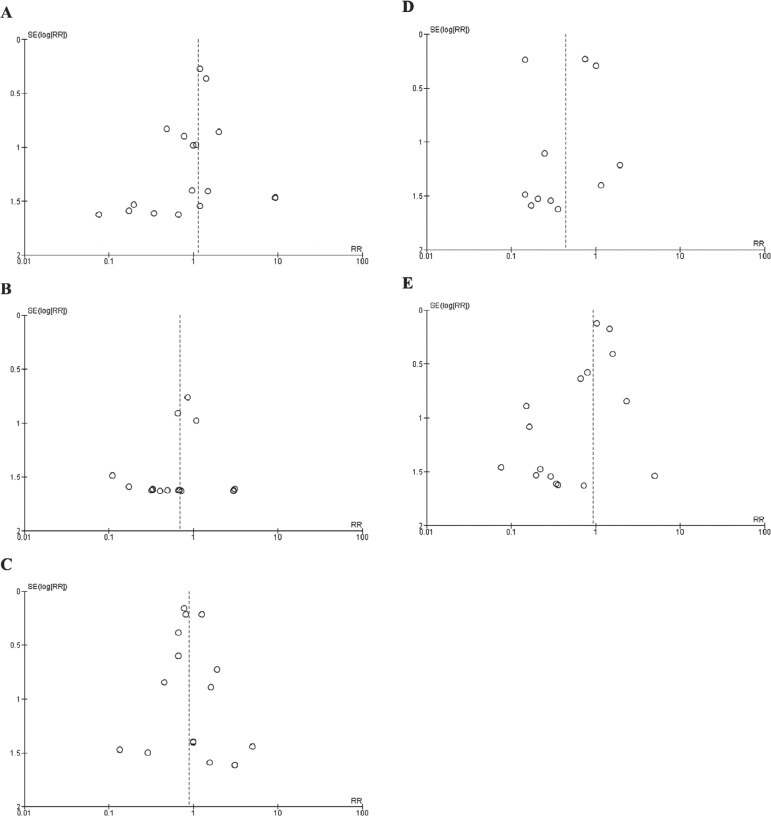
Funnel plots of primary outcomes. Funnel plots for **(A)** stroke, **(B)** systemic embolism, **(C)** all-cause mortality, **(D)** death from cardiogenic causes, and **(E)** major bleeding. Relative risk was used as an effect measure, and standard error was used as a measure of precision. The funnel plots indicated no evidence of publication bias.

Trials of moderate-to-high quality were discerned through the application of the Cochrane method, which involves a comprehensive assessment of critical elements such as randomization, allocation concealment, blinding, and outcome reporting, employed for the scrutiny of RCTs, as depicted in **Supplementary Figure S1**. The assessment using the Newcastle–Ottawa scale unveiled a spectrum of quality within the observational studies included in the analysis, spanning from fair to good, as elucidated in **Supplementary Table S2**. The Newcastle–Ottawa scale is a systematic instrument for evaluating the quality of non-randomized studies—mainly observational studies—by considering selection, comparability, and outcome assessment parameters. The symmetry in the funnel plots instills confidence that the study outcomes remain unaffected by any undue influence of publication bias, as demonstrated in **Supplementary Figure S2**.

### Primary outcomes

The primary outcomes included stroke, systemic embolism, all-cause mortality, cardiac death, and major bleeding.

**Figure 2:**
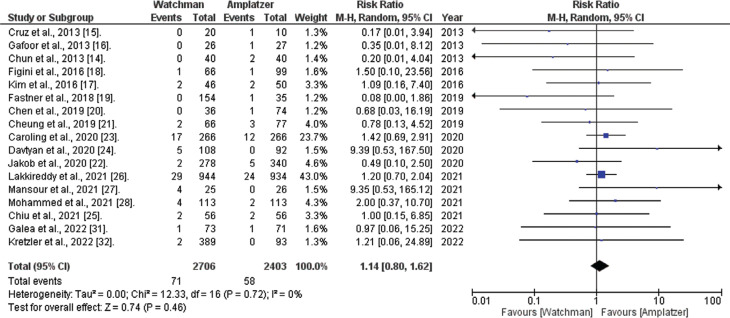
Stroke risk comparison (WATCHMAN™ vs. Amplatzer™ Cardiac Plug). The figure demonstrates that the WATCHMAN™ procedure showed a non-significantly increased risk compared to the Amplatzer™ Cardiac Plug device. *Abbreviations:* CI, confidence interval; M–H, Mantel–Haenszel; RR, relative risk.

*Stroke.* Out of the 20 studies analyzed, 14 studies reported stroke events. The comprehensive analysis revealed that the WATCHMAN™ procedure is associated with a non-significantly increased risk of stroke compared to the ACP method (RR, 1.14; 95% confidence interval [CI], 0.80–1.62; *I*^2^ = 0%; *P* = .46), as depicted in **Figure 2**.

**Figure 3:**
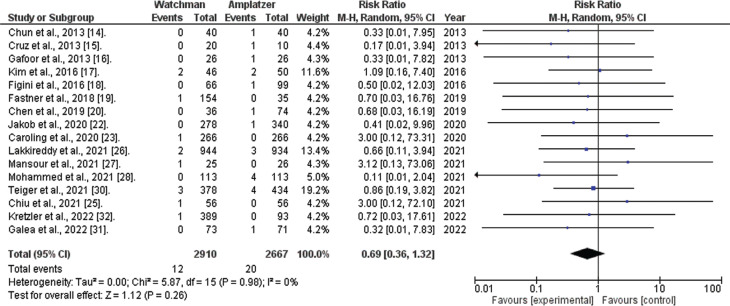
Systemic embolism risk (WATCHMAN™ vs. Amplatzer™ Cardiac Plug). The figure illustrates the combined analysis of 16 studies on systemic embolism occurrence. The analysis indicates that the WATCHMAN™ procedure is associated with a clinically reduced risk of systemic embolism, although statistical significance was not reached. *Abbreviations:* CI, confidence interval; M–H, Mantel–Haenszel; RR, relative risk.

*Systemic embolism.* Data on systemic embolism occurrence were available in 16 of the 20 studies considered. The combined analysis indicated that the WATCHMAN™ procedure is linked to a non-significantly reduced risk of systemic embolism (RR, 0.69; 95% CI, 0.36–1.32; *I*^2^ = 0%; *P* = .26), as depicted in **Figure 3**.

**Figure 4:**
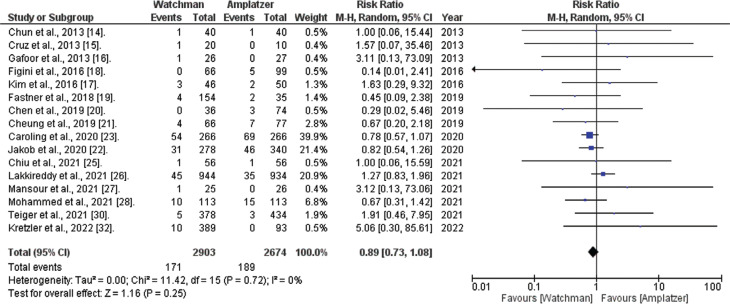
All-cause mortality comparison (WATCHMAN™ vs. Amplatzer™ Cardiac Plug). The figure depicts that the combined analysis suggests that employing the WATCHMAN™ procedure correlates with a clinically diminished risk of all-cause mortality, although statistical significance was not achieved. *Abbreviations:* CI, confidence interval; M–H, Mantel–Haenszel; RR, relative risk.

*All-cause mortality.* Information regarding all-cause mortality was accessible in 16 of the 20 studies included in this study. The combined analysis suggested that the WATCHMAN™ procedure correlates with a non-significantly diminished risk of all-cause mortality (RR, 0.89; 95% CI, 0.73–1.08; *I*^2^ = 0%; *P* = .25), as illustrated in **Figure 4**.

**Figure 5:**
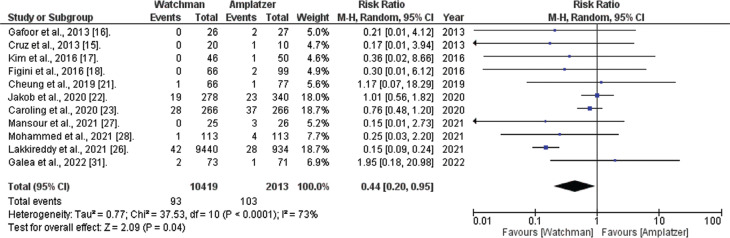
Cardiac death risk (WATCHMAN™ vs. Amplatzer™ Cardiac Plug). The figure presents a comprehensive analysis revealing that the WATCHMAN™ procedure is associated with a significantly lower risk of death from cardiac causes than the Amplatzer™ Cardiac Plug device. *Abbreviations:* CI, confidence interval; M–H, Mantel–Haenszel; RR, relative risk.

*Cardiac death.* Information regarding death from cardiogenic causes was accessible in 11 of the 20 studies evaluated. The comprehensive analysis showed that the WATCHMAN™ procedure is linked to a significantly lower risk of cardiac death compared to the ACP procedure (RR, 0.44; 95% CI, 0.20–0.95; *I*^2^ = 73; *P* = .04), as shown in **Figure 5**.

**Figure 6:**
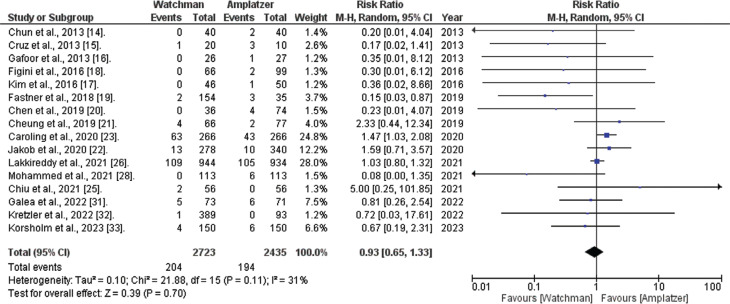
Major bleeding risk (WATCHMAN™ vs. Amplatzer™ Cardiac Plug). The figure depicts that the combined analysis suggests that the WATCHMAN™ procedure is associated with a clinically reduced risk of major bleeding compared to the Amplatzer™ Cardiac Plug device; however, statistical significance was not attained. *Abbreviations:* CI, confidence interval; M–H, Mantel–Haenszel; RR, relative risk.

*Major bleeding.* Data about significant bleeding events were available in 16 of the 20 included studies. The combined analysis indicated that the WATCHMAN™ procedure is associated with a non-significantly reduced risk of major bleeding compared to the ACP procedure (RR, 0.93; 95% CI, 0.65–1.33; *I*^2^ = 31%; *P* = .70), as illustrated in **Figure 6**.

### Secondary outcomes

The secondary outcomes included device-related thrombus, PDL, procedure-related complications, and procedural success. Fourteen out of 20 studies provided information on device-related thrombus, and the pooled analysis indicated that the WATCHMAN™ procedure was associated with a non-significantly increased risk of device-related thrombus (RR, 1.38; 95% CI, 0.82–2.33; *P* = .22). Thirteen out of 20 studies reported data on PDL, revealing a non-significantly increased risk associated with the WATCHMAN™ procedure (RR, 1.52; 95% CI, 0.82–2.81; *P* = .18). Fifteen out of 20 studies reported data on procedure-related complications, demonstrating a non-significantly decreased risk with the WATCHMAN™ procedure (RR, 0.71; 95% CI, 0.47–1.08; *P* = .11). Nine out of 20 studies provided data regarding procedural success, showing no significant difference between the WATCHMAN™ procedure and ACP device groups (RR, 1.04; 95% CI, 0.96–1.13; *P* = .37) in the pooled analysis.

## Discussion

This systematic review and meta-analysis of the WATCHMAN™ and ACP/Amulet™ devices for percutaneous LAAC consisting of 6310 patients from 20 different studies found numerous essential findings. First, the WATCHMAN™ device was associated with an increased stroke risk and a clinically reduced systemic embolism risk, but these differences were not statistically significant. However, the most recent research done by Tan et al.^35^ has yielded extensive insights into the feasibility and efficacy of the WATCHMAN™ device as a substitute for stroke prevention in specific patient populations. Unlike standard anticoagulant therapies for non-valvular AF, these studies suggest that the WATCHMAN™ device may reduce bleeding duration and, in most cases, carry a stroke risk that is either equivalent to or lower than that of conventional anticoagulants.^36,37^

Per our analysis, the WATCHMAN™ device reduced all-cause mortality, although without statistical significance. Moreover, it significantly lowered the risk of cardiac death compared to the ACP device. Despite a decrease in the likelihood of severe bleeding with WATCHMAN™ treatment, the association lacked statistical significance. Yerasi et al. conducted a study summarizing the Amulet™ multicenter registry findings, revealing a procedure fatality rate of 0.2%. The procedure fatality rate in the U.S. LARIAT multicenter registry was notably lower, at 0.006%.^37^ Noteworthy findings from the WATCHMAN™ Left Atrial Appendage System for Embolic Protection in Patients with Atrial Fibrillation (PROTECT-AF) trial indicated that severe bleeding, according to Bleeding Academic Research Consortium (BARC) criteria, occurred exclusively in the pericardial area, with a frequency of 4.8%. In the Amulet™ multicenter registry, significant bleeding occurred in 2.4% of patients, with pericardial bleeding accounting for half of these. It is worth noting that access site bleeding, while relatively infrequent across all devices, occurred in 0.3% of patients.^37^ Secondary findings indicated that the WATCHMAN™ approach could increase device-related thrombus and leaks near the device, although statistical significance was not observed. The WATCHMAN™ device showed a statistically insignificant decrease in the likelihood of complications connected to the surgery while maintaining a procedural success rate comparable to the ACP. The results of our study reveal subtle variations in the safety and effectiveness characteristics of the WATCHMAN™ and ACP/Amulet™ systems. The WATCHMAN™ device demonstrates certain therapeutic benefits. However, many of these findings still need to reach statistical significance.

Appendages with proximal lobes, large ostia, or a shallow depth may make complete sealing difficult and result in PDLs. Furthermore, it has been demonstrated that poor use of the LAAO device increases the risk of PDLs.^38^ It has been shown that individuals with PDLs are more likely to have recurrent thromboembolism or require continuing oral anticoagulant medication, which can diminish LAAO therapeutic efficacy. Physicians usually recommend oral anticoagulants to patients with severe PDLs until the leak size is reduced or a secondary surgery is performed to occlude, coil, or ablate the leak.^39^

Our research is subject to specific limitations. First, few studies provide information on neck diameter, fluoroscopy duration, residual flow, device size, LAA diameter, or procedural time that might be used to choose the suitable device for LAAO. Additionally, the diverse follow-up periods (ranging from 45 days to 4 years) between studies may have had different effects on the observed outcomes. More extended follow-up periods might have revealed cumulative or delayed effects. In contrast, shorter follow-up periods might have captured early effects.

Moreover, the results may be affected, and their applicability is limited by differences in baseline variables, including age, average CHA_2_DS_2_-VASc score, HAS-BLED score, comorbidities, and therapeutic interventions. Additionally, the high rates of cardiac death, major bleeding, and incomplete or inadequately reported data in some studies make it difficult to conduct a thorough analysis and draw reliable conclusions. These factors and differences in study designs, patient populations, and methodology among the included studies introduce heterogeneity in some outcomes.

## Conclusion

Overall, the use of WATCHMAN™ devices has demonstrated a higher likelihood of stroke and a lower likelihood of systemic embolism, all-cause mortality, cardiac death, and major bleeding, respectively. However, these findings were not statistically significant and, therefore, cannot be clinically applied to determine whether WATCHMAN™ devices are superior or inferior to Amplatzer™ Amulet™ devices. The only statistically significant difference was observed in the risk of cardiac death, which may have clinical implications. Additional RCTs are necessary to comprehensively compare the two devices, facilitating better-informed decision-making for cardiologists in the future.

## Figures and Tables

**Table 1:** Baseline Characteristics

Study and Year	Type of Study	Follow-up Duration	Total Number of Participants	Participants in the WATCHMAN™ Group	Participants in the Amulet™ Group	Age (Years)		Male Sex		CHA_2_DS_2_-VASc Score (Points) (Mean ± SD)		HAS-BLED Score (Points)	
						WATCHMAN™	Amulet™	WATCHMAN™	Amulet™	WATCHMAN™	Amulet™	WATCHMAN™	Amulet™
Chun et al. (2013)^15^	Prospective observational	12 months	80	40	40	76 ± 8	76 ± 9	22 (55%)	24 (60%)	4.1 ± 1.5	4.5 ± 1.8	3.1 ± 1.1	3.1 ± 1.2
Cruz-Gonzalez et al. (2014)^16^	Prospective observational	3 months	30	20	10	76 ± 8.2	77.49 ± 5.4	7 (33%)	5 (50%)	3.4 ± 0.7	3.7 ± 0.823	4.2 ± 4	4.7 ± 1.25
Gafoor et al. (2014)^17^	Retrospective observational	12 months	53	26	27	83 ± 2.6	83.9 ± 3.0	14 (53.80%)	19 (70.40%)	5.3 ± 1.4	5.0 ± 1.5	N/A	N/A
Kim et al. (2016)^18^	Retrospective observational	6 months	96	46	50	64.7 ± 10.0	65.1 ± 9.4	27	32	4.1 ± 1.7	3.6 ± 1.6	2.8 ± 1.2	2.7 ± 1.3
Figini et al. (2017)^19^	Retrospective observational	12 months	165	66	99	72 ± 8	72 ± 9	38	71	3.8 ± 1.6	4 ± 1.7	3.4 ± 1.3	3.7 ± 1.5
Fastner et al. (2018)^20^	Retrospective observational	6 months	189	154	35	75.2 ± 2.8	77.1 ± 9.7	105 (68.2%)	22 (62.9%)	4.5 ± 0.1	4.0 ± 1.4	3.6 ± 0.2	3.7 ± 1.0
Chen et al. (2019)^21^	Prospective observational	6 months	110	36	74	75.3 ± 8.8	76.0 ± 7.9	26	50	3.6 ± 1.5	3.9 ± 1.5	3.8 ± 1.0	3.9 ± 1.1
Cheung et al. (2019)^22^	Retrospective observational	28 months	143	66	77	72.6 ± 7.9	71 ± 8.6	45 (68.2%)	50 (64.9%)	4.3 ± 1.4	3.9 ± 1.7	3.1 ± 1	2.7 ± 1.1
Ledwoch et al. (2020)^23^	Observational	12 months	618	278	340	N/A	N/A	N/A	N/A	4.4 ± 1.5	4.6 ± 1.6	3.9 ± 1.0	3.9 ± 1.2
Kleinecke et al. (2020)^24^	Observational	30 months	532	266	266	N/A	N/A	N/A	N/A	4.5 ± 1.7	4.5 ± 1.5	3.2 ± 1.0	3.2 ± 1.0
Davtyan et al. (2020)^25^	Prospective observational	12 months	200	108	92	66.9 ± 7.9	66.7 ± 7.6	48 (44.60%)	43 (46.50%)	3.5 ± 1.6	4 ± 1.6	3 ± 2.1	3 ± 0.9
Chiu et al. (2022)^26^	Retrospective observational	12 months	112	56	56	72.7 ± 8.3	71.1 ± 11.4	32 (57%)	36 (64%)	4.2 ± 1.4	3.9 ± 1.8	3.5 ± 1.7	3.3 ± 1.9
Lakkireddy et al. (2021)^27^	RCT	18 months	1878	944	934	75.1 ± 7.6	75.0 ± 7.6	579 (61.3%)	549 (58.8%)	4.7 ± 1.4	4.5 ± 1.3	3.3 ± 1.0	3.2 ± 1.0
Mansour et al. (2022)^28^	RCT	12 months	51	25	26	76 ± 6.9	75 ± 7.4	19 (76%)	20 (76%)	3.9 ± 1.27	3.9 ± 1.16	4.2 ± 0.9	4.1 ± 1.2
Saad et al. (2021)^29^	Retrospective observational	6–8 months	226	113	113	77 ± 2.5	78 ± 2	70 (62%)	70 (62%)	4 ± 2.3	4 ± 2.5	3 ± 1.1	3 ± 1.4
Radinovic et al. (2021)^30^	Non-RCT	4 years	224	99	125	N/A	N/A	N/A	N/A	3.5 ± 0.6	3.3 ± 0.9	4.1 ± 1.2	3.9 ± 0.5
Teiger et al. (2021)^31^	Prospective observational	16 months	812	378	434	75.5 ± 0.3	75.5 ± 0.3	N/A	N/A	4.6 ± 0.1	N/A	3.2 ± 0.05	N/A
Galea et al. (2022)^32^	Prospective observational	45 days	144	73	71	77.1 ± 7.3	76.5 ± 8.4	50 (68.5%)	51 (71.8%)	4.4 ± 1.4	4.2 ± 1.4	3.2 ± 1.0	3.1 ± 0.8
Kretzler et al. (2022)^33^	Prospective observational	6 weeks	482	389	93	N/A	N/A	256 (66%)	56 (58%)	4.1 ± 1.5	4.2 ± 1.5	3.6 ± 1.1	3.3 ± 1.2
Korsholm et al. (2023)^34^	Retrospective observational	18 months	300	150	150	74.2 ± 8.3	73.4 ± 9.6	115 (76.6%)	103 (68.6%)	4.1 ± 1.6	3.9 ± 1.5	2.5 ± 1.0	2.7 ± 1.0

*Abbreviations:* N/A, not available; RCT, randomized controlled trial.

**Supplementary Table S1:** Search Strategy

Database	Search Strategy	Results
PubMed	((“Watchman”[All Fields] AND (““device s”[All Fields] OR “equipment and supplies”[MeSH Terms] OR (“equipment”[All Fields] AND “supplies”[All Fields]) OR “equipment and supplies”[All Fields] OR “device”[All Fields] OR “instrumentation”[MeSH Subheading] OR “instrumentation”[All Fields] OR “devices”[All Fields])) OR (“Watchman”[All Fields] AND (“closure”[All Fields] OR “closure s”[All Fields] OR “closures”[All Fields])) OR (“Watchman”[All Fields] AND (“drug implants”[MeSH Terms] OR (“drug”[All Fields] AND “implants”[All Fields]) OR “drug implants”[All Fields] OR “implant”[All Fields] OR “embryo implantation”[MeSH Terms] OR (“Left”[All Fields] AND (“atrial appendage”[MeSH Terms] OR (“atrial”[All Fields] AND “appendage”[All Fields]) OR “atrial appendage”[All Fields]) AND (“dental occlusion”[MeSH Terms] OR (“dental”[All Fields] AND “occlusion”[All Fields]) OR “dental occlusion”[All Fields] OR “occlusion”[All Fields] OR “occlused”[All Fields] OR “occlusions”[All Fields] OR “occlusive”[All Fields] OR “occlusives”[All Fields]) AND (“device s”[All Fields] OR “equipment and supplies”[MeSH Terms] OR (“equipment”[All Fields] AND “supplies”[All Fields]) OR “equipment and supplies”[All Fields] OR “device”[All Fields] OR “instrumentation”[MeSH Subheading] OR “instrumentation”[All Fields] OR “devices”[All Fields]))) AND (((“amulet”[All Fields] OR “amulets”[All Fields]) AND (“device s”[All Fields] OR “equipment and supplies”[MeSH Terms] OR (“equipment”[All Fields] AND “supplies”[All Fields]) OR “equipment and supplies”[All Fields] OR “device”[All Fields] OR “instrumentation”[MeSH Subheading] OR “instrumentation”[All Fields] OR “devices”[All Fields])) OR ((“amulet”[All Fields] OR “amulets”[All Fields]) AND (“closure”[All Fields] OR “closure s”[All Fields] OR “closures”[All Fields])) OR ((“amulet”[All Fields] OR “amulets”[All Fields]) AND (“drug implants”[MeSH Terms] OR (“drug”[All Fields] AND “implants”[All Fields]) OR “drug implants”[All Fields] OR ((“left atrial appendage closure”[MeSH Terms] OR (“Left”[All Fields] AND “atrial”[All Fields] AND “appendage”[All Fields] AND “closure”[All Fields]) OR “left atrial appendage closure”[All Fields]) AND (“device s”[All Fields] OR “equipment and supplies”[MeSH Terms] OR (“equipment”[All Fields] AND “supplies”[All Fields]) OR “equipment and supplies”[All Fields] OR “device”[All Fields] OR “instrumentation”[MeSH Subheading] OR “instrumentation”[All Fields] OR “devices”[All Fields]))) AND (“left atrial appendage closure”[MeSH Terms] OR (“Left”[All Fields] AND “atrial”[All Fields] AND “appendage”[All Fields] AND “closure”[All Fields]) OR “left atrial appendage closure”[All Fields] OR (“left atrial appendage closure”[MeSH Terms] OR (“Left”[All Fields] AND “atrial”[All Fields] AND “appendage”[All Fields] AND “closure”[All Fields]) OR “left atrial appendage closure”[All Fields] OR (“laa”[All Fields] AND “closure”[All Fields]) OR “laa closure”[All Fields]) OR (“Left”[All Fields] AND (“atrial appendage”[MeSH Terms] OR (“atrial”[All Fields] AND “appendage”[All Fields]) OR “atrial appendage”[All Fields]]	1500
Elsevier’s ScienceDirect	(Watchman device OR Watchman closure OR Watchman implant OR left atrial appendage occlusion device) AND (Amulet device OR Amulet closure OR "Amulet implant OR left atrial appendage closure device) AND (Left atrial appendage closure OR LAA closure OR Left atrial appendage occlusion) AND (Atrial fibrillation OR AFib) AND (Clinical effectiveness OR “Clinical outcomes OR Efficacy OR Effectiveness) AND (Safety assessment OR Safety profile OR Complications)	950
Cochrane Library	(Watchman device OR Watchman closure OR Watchman implant OR left atrial appendage occlusion device) AND (Amulet device OR Amulet closure OR “Amulet implant OR left atrial appendage closure device) AND (Left atrial appendage closure OR LAA closure OR Left atrial appendage occlusion) AND (Atrial fibrillation OR AFib) AND (Clinical effectiveness OR “Clinical outcomes OR Efficacy OR Effectiveness) AND (Safety assessment OR Safety profile OR Complications)	550

*Abbreviation:* MeSH, Medical Subject Heading.

**Supplementary Table S2:** The Newcastle–Ottawa Scale

Study	Selection				Comparability	Outcomes			Total
	Representativeness of the Exposed Cohort	Selection of the Non-exposed Cohort	Ascertainment of Exposure	Demonstration that Outcome of Interest Was Not Present at Start of Study	Comparability of Cohorts on the Basis of the Design or Analysis	Assessment of Outcome	Was Follow-up Long Enough for Outcomes to Occur?	Adequacy of Follow-up of Cohorts	
Chun et al. (2013)^15^	*	*	*	*	**	*	*	*	*********
Cruz-Gonzalez et al. (2014)^16^	*	*	*	*	*	*	*	*	********
Gafoor et al. (2014)^17^	*	*	*	*	*	*	*	*	********
Kim et al. (2016)^18^	*	*	*	−	*	*	*	*	******
Figini et al. (2017)^19^	*	*	*	*	**	*	*	*	*********
Fastner et al. (2018)^20^	*	*	*	*	*	*	*	*	********
Chen et al. (2019)^21^	*	*	*	*	*	*	*	*	********
Cheung et al. (2019)^22^	*	*	*	−	*	*	*	*	******
Ledwoch et al. (2020)^23^	*	*	*	*	**	*	*	*	*********
Kleinecke et al. (2020)^24^	*	*	*	*	*	*	*	*	********
Davtyan et al. (2020)^25^	*	*	*	−	*	*	*	*	******
Chiu et al. (2022)^26^	*	*	*	*	**	*	*	*	*********
Saad et al.(2021)^29^	*	*	*	*	*	*	*	*	********
Radinovic et al.(2021)^30^	*	*	*	−	*	*	*	*	******
Teiger et al. (2021)^31^	*	*	*	*	**	*	*	*	*********
Galea et al. (2022)^32^	*	*	*	*	*	*	*	*	********
Kretzler et al. (2022)^33^	*	*	*	−	*	*	*	*	******
Korsholm et al. (2023)^34^	*	*	*	*	**	*	*	*	*********

This table thoroughly assesses the quality of observational studies incorporated into the meta-analysis, using the Newcastle–Ottawa scale. The evaluation delineates a spectrum of study quality, ranging from fair to good. In the Newcastle–Ottawa scale, an asterisk (*) signifies a point awarded for meeting specific criteria, with good quality defined as 3 or 4 stars in the selection domain, 1 or 2 stars in the comparability domain, and 2 or 3 stars in the outcome/exposure domain. Fair quality is characterized by 2 stars in the selection domain, 1 or 2 stars in the comparability domain, and 2 or 3 stars in the outcome/exposure domain. Poor quality is indicated by 0 or 1 star(s) in the selection domain, 0 stars in the comparability domain, or 0 or 1 star(s) in the outcome/exposure domain.
